# Selection of Effective Antibiotics for Uropathogenic *Escherichia coli* Intracellular Bacteria Reduction

**DOI:** 10.3389/fcimb.2020.542755

**Published:** 2020-10-21

**Authors:** María José González, Pablo Zunino, Paola Scavone, Luciana Robino

**Affiliations:** ^1^ Departamento de Microbiología, Instituto de Investigaciones Biológicas Clemente Estable, Montevideo, Uruguay; ^2^ Departamento de Bacteriología y Virología, Facultad de Medicina, Universidad de la República, Montevideo, Uruguay

**Keywords:** intracellular bacteria, uropathogenic *E. coli*, antibiotics, urinary tract infection, recurrent infection

## Abstract

Urinary tract infections (UTI) are one of the most frequent bacterial infections in humans, being Uropathogenic *Escherichia coli* (UPEC), the most common etiological agent. The ability of UPEC to invade urothelial cells and to form intracellular bacterial communities (IBC) has been described. Therefore, UPEC can persist in the urinary tract producing recurrent infections, resisting antibiotic activity. The objective of the present work was to analyze the ability of a collection of UPEC clinical isolates to invade bladder epithelial cells in vitro and the activity of different classes of antibiotics on intracellular bacteria. We selected 23 UPEC clinical isolates that had been previously detected intracellularly in desquamated bladder epithelial cells from patients’ urine. A cellular invasion assay using the T24 bladder cell line was used. Intracellular bacteria was confirmed by laser confocal microscopy. All the strains were able to invade the cells with different percentages of intracellular bacterial survival (0.7 to 18%). However, no significant relationship was found between the percentage of in vitro infection and the presence of IBC in desquamated urine cells. In vitro, intracellular bacteria were confirmed in four representative strains by confocal laser microscopy. Ceftriaxone, ciprofloxacin and, azithromycin in vitro activity on intracellular bacteria were evaluated. Amikacin was used as a negative control. All the antibiotics tested, except amikacin, significantly decreased the number of intracellular bacteria. Ciprofloxacin was the antibiotic that induced the highest decrease percentage. Conclusions: All UPEC clinical isolates could invade bladder epithelial cells in vitro. Ceftriaxone, ciprofloxacin, and azithromycin can reduce the percentage of intracellular bacteria in vitro. In vivo studies are needed to confirm the utility of these antibiotics for intracellular bacteria reduction in UTI.

## Introduction

Urinary tract infections (UTI) are among the most common bacterial infections in humans. At least 40% of woman, 12 % of men, and 5% of children would have one episode of UTI in their life (O’Hanley, 1996; Habib, 2012). Following an initial UTI, approximately 25% of women and 20–40% of children will experience a recurrent UTI episode within the following 12 months. (O’Hanley, 1996; Garin et al., 2006; Conway et al., 2007).

Uropathogenic *Escherichia coli* (UPEC) is the most common etiological agent, being responsible for about 75% of the cases (Flores-Mireles et al., 2015).

UPEC from the intestinal microbiota can colonize the perineum and ascend through the urethra to reach the bladder epithelium. In the bladder, *E. coli* adheres to the uroepithelium through fimbriae, eliciting an inflammatory response responsible for cystitis symptoms (Sobel and Kaye, 2005). It has been demonstrated that UPEC can invade the superficial bladder epithelial cells resulting in the formation of intracellular bacterial communities (IBC) or quiescent intracellular reservoirs (QIR) within immature bladder epithelial cells (Flores-Mireles et al., 2015). The FimH adhesin of type 1 pili recognizes uroplakins and integrins receptors on the umbrella surface cells of the bladder epithelium (Wright and Seed, 2007). After binding, actin filaments rearrange by activation of the RHO GTPases family (such as those of the Rac family), and bacterial endocytosis occurs (Eto et al., 2007). Once internalized, bacteria can be expelled from the cells by exocytosis or evade this innate defense mechanism, enter into the cytoplasm and multiply forming IBC (Song et al., 2009).

Different authors have described the dynamics of IBC formation in a murine model (Mulvey et al., 2001; Justice et al., 2004; Schwartz et al., 2011). Six hours after infection, an early IBC is formed, constituted by a group of scarce coccoid bacteria. In the following 6 h, the IBC expands, and bacteria are tightly grouped (intermediate IBC). Anderson et al. described IBC at 24 h post-infection in superficial umbrella cells (Anderson et al., 2003). At 16–24 h, bacteria are released from the late IBC, adopting a filamentous morphology and can emerge from the host cell (Mulvey et al., 2001; Schwartz et al., 2011). Bacteria in the bladder lumen can invade new superficial epithelial cells and initiate a new cycle of IBC formation. The inflammatory process and polymorphonuclear recruitment stimulate apoptosis and exfoliation of the superficial urothelium cells, exposing deeper immature cells to UPEC. In the early stages of infection, HlyA (toxin alfa-hemolysin) could trigger exfoliation, and deeper layers of the uroepithelium are exposed to be invaded by UPEC (Nagamatsu et al., 2015). Therefore, bacterial access is facilitated, and QIRs are developed (Mulvey et al., 2001). In the other hand CNF-1 (cytotoxic necrotizing factor-1) activates RAC-1 that has anti-apoptotic and pro-survival pathways preventing the apoptosis of the colonized epithelial cells and allowing UPEC to survive intracellularly (Garcia et al., 2013)

In humans, Rosen et al. and Robino et al. described the presence of IBC in desquamated epithelial cells in urine from patients with acute UTI (women and children, respectively) (Rosen et al., 2007; Robino et al., 2014). The presence of IBC was evidenced in 18% of urines from women with cystitis and 36.8% of urine samples from children with UTI (Robino et al., 2014). It is proposed that IBC could be related to host defenses evasion, antibiotic resistance, and recurrent UTI. In children with UTI, the presence of IBC was statistically associated with recurrent UTI (Robino et al., 2014).

However, some questions arise: do all UPEC clinical isolates invade urothelial cells and form IBC? Does IBC occur in all UTI episodes caused by UPEC?

Clinical Guidelines suggest the use of different families of antibiotics for UTI treatment, such as beta-lactams, quinolones, fosfomycin, nitrofurantoin, and aminoglycosides (Urinary tract infection: diagnosis, treatment and long-term management of urinary tract infection in children, 2007; Gupta et al., 2011). However, only quinolones achieve high intracellular concentrations. So, if bacteria invade epithelial cells, are these antibiotics able to eliminate intracellular bacteria?

The aim of the present study was to analyze the ability of a collection of UPEC clinical isolates to invade bladder epithelial cells “in vitro” and the activity of different classes of antibiotics on intracellular bacteria.

## Materials and Methods

### Bacterial Strains

In an earlier investigation, from our group of work, urine specimens from 133 children with *E. coli* UTI were studied for the presence of intracellular bacteria (IB) in desquamated bladder epithelial cells by confocal laser microscopy (Robino et al., 2014).

The presence of IB was detected in 49 of 133 (36.8%) samples by confocal microscopy, in 30 cases as IBC, and in 19 as isolated intracellular bacteria (IIB) (Robino et al., 2014). Phylogenetic group, virulence factors, and biofilm production were assessed in this bacterial collection (Robino et al., 2014; González et al., 2017). For the present work, 23 *E. coli* (from different phylogenetic groups and virulence factors), were selected from this collection of pediatric clinical isolates (9 strains from the IBC group, 5 from the IIB group, and 9 from no IBC or IIB group).

The characteristic of the selected strains is shown in **Table 1**.

**Table 1:** Selected UPEC strains to evaluate intracellular bacterial invasion in vitro.

	IBC	IIB	Phylogenetic group	papA	papC	sfa/focDe	afa/draBC	kpsMTII	iutA	fimH	papEF	papGIII	sfaS	KpsMT k1	KpsMT k5	hlyA	cnf1	ibeA	PAI	fyuA	ATB susceptibility	BF
7	+	–	B2	–	–	+	–	+	–	+	–	+	–	+	–	–	–	–	+	+	ALL^s^	No
145	+	–	A	–	–	–	+	+	+	+	–	–	–	–	–	–	–	–	–	+	ALL^s^	No
98	+	–	A	–	–	–	–	–	–	+	–	+	+	–	–	–	–	–	–	–	ALL^s^	No
121	+	–	D	–	–	–	–	–	+	+	–	+	+	–	–	–	–	–	–	–	ALL^s^	No
155	+	–	B2	+	+	–	–	–	+	+	+	+	–	+	–	–	–	–	–	+	ALL^s^	Yes
198	+	–	D	–	–	–	–	–	–	+	+	+	–	–	–	–	–	–	+	+	ALL^s^	No
208	+	–	D	–	–	–	–	+	–	+	–	+	–	–	–	–	–	–	–	+	ALL^s^	No
221	+	–	D	+	+	–	+	+	+	+	+	–	–	+	–	–	–	–	+	+	ALL^s^	No
230	+	–	B1	–	–	–	–	–	–	+	–	–	+	–	–	–	–	–	–	–	ALL^s^	Yes
13	–	+	D	–	–	–	+	+	+	+	–	–	–	+	–	–	–	–	–	+	ALL^s^	No
144	–	+	B1	–	–	–	–	+	–	+	–	–	+	–	–	–	–	–	–	–	ALL^s^	Yes
173	–	+	A	–	–	–	+	+	+	–	–	–	–	–	–	–	–	–	–	–	ALL^s^	Yes
182	–	+	D	–	–	–	–	+	–	+	–	+	–	+	–	–	–	–	–	+	ALL^s^	No
156	–	+	B1	–	–	–	–	+	–	+	–	+	–	–	–	–	–	–	–	–	ALL^s^	No
30	–	–	D	+	+	–	–	+	+	+	–	–	–	+	–	–	–	–	+	+	ALL^s^	No
95	–	–	B2	+	+	+	–	+	+	+	+	–	–	+	–	–	–	–	+	+	AK^I^	Yes
172	–	–	B2	–	–	+	+	+	+	+	–	–	–	–	+	+	–	–	–	–	ALL^s^	No
174	–	–	B2	+	+	+	+	+	+	+	+	+	+	–	–	–	+	–	–	–	ALL^s^	No
191	–	–	B2	+	+	+	–	+	–	+	–	+	–	–	+	+	+	–	+	+	ALL^s^	No
194	–	–	B2	+	+	–	–	+	–	+	–	–	–	–	+	–	–	–	+	+	ALL^s^	No
226	–	–	B2	+	+	–	–	+	+	+	–	–	–	–	+	–	–	–	–	+	ALL^s^	No
234	–	–	B2	–	–	+	–	+	–	+	+	–	–	–	+	–	+	–	–	+	ALL^s^	No
237	–	–	A	–	–	–	–	–	–	+	–	+	–	–	–	–	–	–	–	–	ALL^s^	Yes

IBC, intracellular bacterial community; IIB, isolated intracellular bacteria; papa, papC, papEF, and papGIII, P pili; sfa/focDE, S and Dra fimbriae; afa/draBC, afimbrial adhesin; KPSMTII, type II capsule; K1 and K5, capsule; iutA, siderophore aerobactin; FimH, type 1 pili; sfaS, S fimbriae; hlyA, hemolysin A; cnf1, toxin; ibeA, invasin; PAI, pathogenicity island; fyuA, siderophore; ND, no determined; ATB, antibiotic; S, susceptible; I, intermediate; R, resistant; NAL, nalidixic acid; AK, amikacyn; BF, Biofilm. Strains highlighted in gray are the strains selected for antibiotic effect on IBC assay.

Antibiotic susceptibility to gentamicin, amikacin, ceftriaxone, ciprofloxacin, and azithromycin was detected using VITEKⓇ 2 Compact System (bioMerieux, Marcy-l’E´ toile, France) and results were interpreted following Clinical and Laboratory Standards Institute (CLSI) recommendations (CLSI, 2018). All the strains included were susceptible to these antibiotics (MIC for ceftriaxone ≤ 1 µg/ml, ciprofloxacine ≤ 1 µg/ml, and azithromycin ≤16 µg/ml).

### Bacterial Invasion Assay

Bacterial invasion assay was performed according to Blango et al. and Lyer et al. with some modifications, using the T24 cell line derived from human bladder transitional epithelium carcinoma (ATCC HTB-4) (Blango and Mulvey, 2010; Scavone et al., 2015; Lyer et al., 2018). The human bladder epithelial cell line T24 (ATCC HTB-4) was maintained at 37°C and 5% CO2 in DMEM medium supplemented with 10% heat-inactivated fetal bovine serum (Gibco). Before each experiment, the number of eukaryotic cells was counted using a Neubauer chamber in order to set up the number of bacteria to reach the desired multiplicity of infection (MOI). The bacterial strains were grown statically at 37°C for 24 h in Luria Bertani (LB) broth to induce Type 1 pilus expression (Blango and Mulvey, 2010). T24 cell monolayers were infected with the bacterial suspensions in DMEM using an MOI of 15 bacteria per host cell. The initial inoculum was quantified through plate count on LB Agar during 24 h, 37°C. After 2 h incubation of cells with bacteria at 37°C and 5% CO2, the cells were washed three times with PBS containing Ca2+ and Mg2+ (PBS2) to remove any non-adherent bacteria (Blango and Mulvey, 2010). Monolayers were then incubated for another 2 h with complete DMEM medium plus 100 µg/ml of gentamicin to kill extracellular bacteria. After additional washes with PBS2 were performed, cells were incubated for other 18 h with DMEM containing a lower concentration of gentamicin (10 µg/ml) (Blango and Mulvey, 2010). Lastly, after 3 washes with PBS, monolayer cells were lysed with PBS supplemented with 0.4% Triton X-100 and 1/10 serial dilutions were cultivated in LB Agar and incubated at 37°C for 18–24 h for colony count (Blango and Mulvey, 2010). The intracellular bacterial survival percentage was calculated as (CFUf/CFUi) x 100 been CFUf the values of CFU recovered intracellular after the incubation period and CFUi the initial inoculum for each strain. The assay and the plate count were performed in triplicate for each strain.

### Visualization of Intracellular Bacteria by Confocal Laser Microscopy

From the 23 strains, four (7, 144, 172, and 174) with the highest invasion percentage and previously characterized in human urine (**Table 1**) as IBC producers (1 strain), IIB (1 strain) or not IBC producers (2 strains) (Robino et al., 2014) were selected to confirm the localization of bacteria within the T24 cells.

The cellular invasion assay was performed as described above (see bacterial invasion assay), although in this case the cellular monolayer was grown on a coverslip. After the entire process of intracellular invasion, the culture medium was removed, and the cells were fixed with 4% paraformaldehyde in PBS for 15 min. The immunofluorescence stain on the coverslip started with an initial incubation with no permeabilization (NP) buffer for 20 min (Bovine serum albumin 2%, NH4Cl, 50 mM in PBS Ca/Mg). After washing with PBS, 5.0 µg/ml of Alexa Fluor (R) 350-Wheat Germ Agglutinin (Molecular Probes) staining was performed for 15 min and then cells permeabilization (0,3% Triton X-100 on NP buffer) was performed. After 15 min, slides were washed and incubated with an *E. coli* antibody coupled to FITC (Abcam ab30522) (1/50) for 1 h, at room temperature. Then, the samples were washed with PBS and incubated with Rhodamine Phalloidin, (for actin staining) (Invitrogen) (1/50), Hoescht 33342 (Invitrogen) and 350-WGA (5.0 µg/ml) during 30 min. Once staining was over, the slides were washed with PBS and mounted with 10 µl of ProLong™ Gold Antifade Mountant (Invitrogen™).

Acquisition and processing of 3D image stacks were performed as described before (Schlapp et al., 2011) using an Olympus BX-61 FV300 CLS microscope and 350/460, 488/520, and 543/565 excitation/emission wavelength. The acquisition step size was 0.3 µm in the z-axis and 1024 x 1024 pixels in xy-plane with a pixel size of 70 nm. 3D Image stack was deconvolved with Huygens Scripting Software and were reconstructed using Volocity 3D Image Analysis Software (PerkinElmer).

### Antibiotic Effect on Intracellular Bacteria *In Vitro*

The cellular invasion assay was performed as previously described with the following modifications (Blango and Mulvey, 2010). After finishing the 18 h of incubation, the infected monolayer was incubated for 10 h more with the different antibiotic concentrations. Finally, after 3 washes with PBS, monolayer cells were lysed with PBS plus 0.4% Triton X-100 and 1/10 serial dilutions were cultivated in LB agar and incubated at 37°C for 18–24 h for colony count. The percentage of intracellular survival bacteria was calculated as it was stated before. Four antibiotics were selected: amikacin at 500, 1,000, and 2,000 µg/ml (this antibiotic was used as a negative control since as gentamicin, it does not penetrate the cellular membrane); ceftriaxone at 500, 1,000, and 1,500 µg/ml, ciprofloxacin at 2, 3, and 5 µg/ml, and azithromycin at 20, 100, and 500 µg/ml. Ceftriaxone and ciprofloxacin are antibiotics commonly used for UTI treatment, and the concentrations selected are those that these antibiotics achieve in urine after a standard dose in humans (Lorian, 2005; Gupta et al., 2011). Azithromycin was selected because of its well-established intracellular activity, although its low urinary excretion makes it unsuitable for UTI treatment (FDA; Gupta et al., 2011). The concentrations were estimated according to the concentration reached in the lung. The results were compared to each control without antibiotics (0 μg/ml). The CFU/ml obtained in infected T24 bladder cells treated with 0 μg/mL of antibiotics was designated 100% of internalized bacteria, and the CFU/ml of all other samples were calculated relative to this value and plotted using GraphPad Prism.

The assay was performed in triplicate with an experimental replica. The Mann-Whitney statistic test was applied, and a p-value ≤0.05 was considered significant.

### Cytotoxicity Assay

Both cellular invasion by bacteria and antibiotics could be cytotoxic. Therefore, the cytotoxicity of bacteria and antibiotics on the cell monolayer was determined in order to avoid confusion when evaluating the effect of antibiotics on intracellular bacteria.

The bacterial strain cytotoxicity was assessed by counting the viable eukaryotic cells after the infection (VC) and compared with an uninfected-control (UC). We calculated the cytotoxicity percentage using the following formula: 100-[(VC/UC)x100].

The antibiotic cytotoxicity alone was determined by incubating the eukaryotic cells during 10 h with the different antibiotic concentrations in 6 wells plates with coverslips. After incubation and 3 washes with PBS, the LIVE/DEAD stain was performed as previously reported by Kabakov et al. (2011) using 5 µg/ml of propidium iodide (PI) and 1 µg/ml of Hoescht 33342. Twenty fields were analyzed for each experiment. Cytotoxicity percentage was calculated as (dead cells/total cells)x100. The results were compared using the Mann-Whitney statistic test, and a p-value ≤0.05 was considered significant.

### Statistical Analyses

All the statistical analyses were performed using GraphPad Prism7 for Mac OS X. The intracellular bacterial survival assay was compared using the Kruskal-Wallis test and was considered significant when p ≤0.05.

The antibiotic effect on intracellular bacteria in vitro, cytotoxicity levels of antibiotics, and UPEC cytotoxicity was compared using the Mann-Whitney non-parametric test, and it was considered significant when p ≤0.05. In order to correlate the cytotoxicity level between the antibiotics and reduction of the intracellular bacteria, Pearson coefficient and correlation matrix were calculated with a significance level of p ≤0.05.

## Results

### Intracellular Bacterial Survival in *In Vitro* Models

To analyze the UPEC intracellular survival capacity, we selected 23 strains previously classified according to the presence/absence of IBC or isolated intracellular bacteria (IIB) in desquamated cells in the urine of children with UTI (Robino et al., 2014).

**Figure 1:**
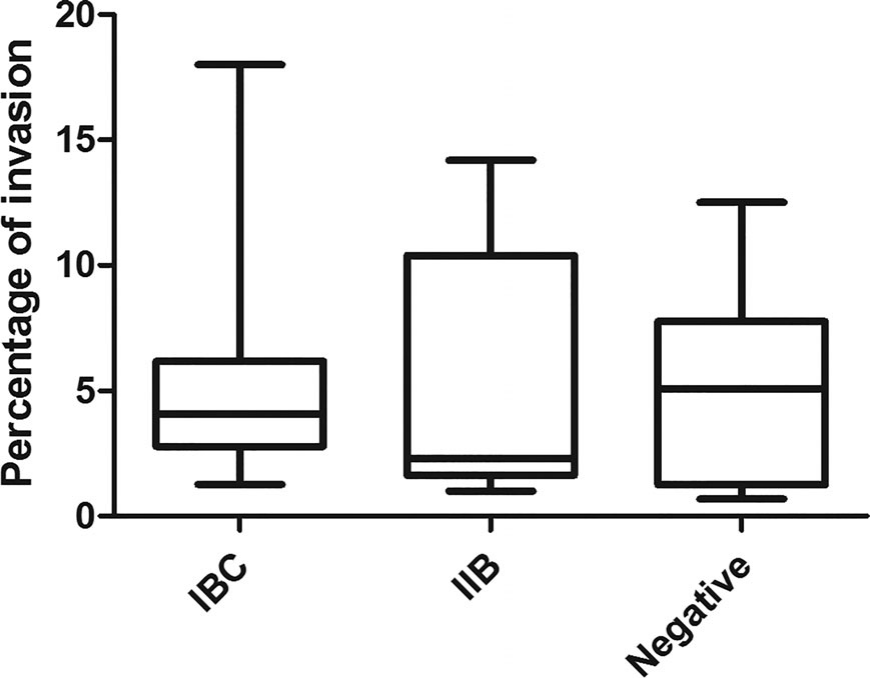
Percentage of UPEC invasion in T24 cells. The intracellular bacterial percentages found in this work and the previous characterization as IBC, IIB, or not detected intracellularly in bladder desquamated cells in children’s urine (Robino et al., 2014) are plotted using graphPrism. Stastistic: Kruskal-Wallis test, p ≤ 0.05 was considered significant. The assay was performed in triplicate for each strain.

Nine out of 23 strains formed IBC, 5 were seen as IIB, and 9 were negative for IBC/IIB. To assess the percentage of intracellular bacteria, plate counting of the viable intracellular bacteria was carried out. The results obtained are shown in **Figure 1**. All the strains were able to invade the cultured cells. The percentages of intracellular bacteria observed varied from 2.6 to 18% in the group previously classified as IBC-producers in humans; 1 to 14.2% in the IIB group and 0.7 to 12.5% ​​in the negative group. No significant differences were observed when the percentages of intracellular bacteria of the different groups (IBC, IIB, and negative) were compared.

**Figure 2:**
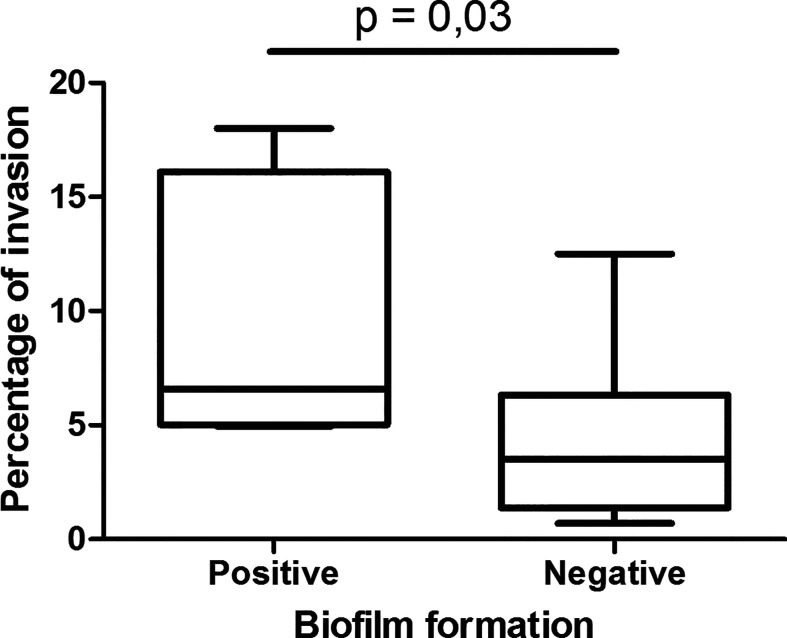
Association between UPEC invasion and biofilm formation. The UPEC strain collection was classified regarding biofilm formation in two categories positive and negative (González et al., 2017). In each group, the distribution of the invasion percentage and its media values were plotted and significant differences were observed using Mann-Whitney statistic test (p-value ≤ 0.05 was considered significant).

UPEC biofilm formation, using crystal violet assay, was previously characterized by our group of work (20). The association of the capacity of the strains to form biofilm and the intracellular bacterial percentages found in this work were analyzed (**Figure 2**). The strains that formed biofilm showed higher invasion percentages, and significant differences were obtained compared to the strains that do not produce biofilm (p 0.03). Another interesting information is that all the strains that produce biofilm and invade epithelial cells express FimH adhesin [hemagglutination assay was performed in a previous work (González et al., 2017)].

The strains that form biofilm showed higher invasion percentages compared with the ones that do not form biofilm.

### UPEC Cytotoxicity on Cells Monolayer

**Figure 3:**
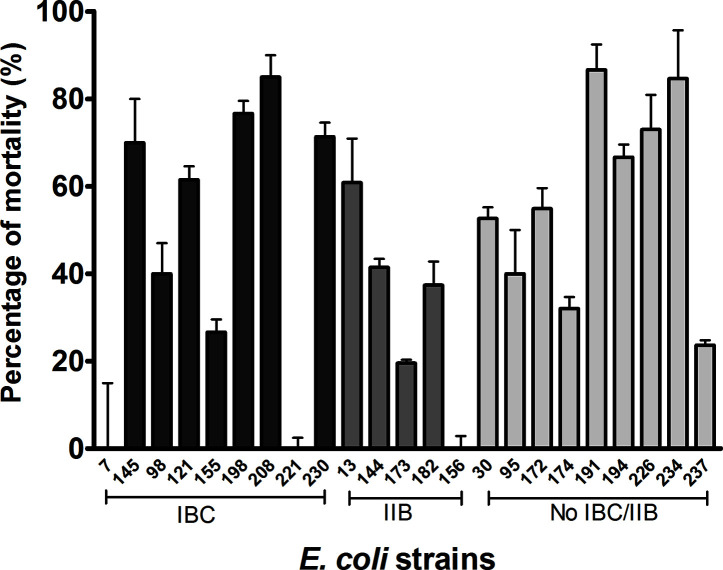
Bacterial cytotoxicity over the cell monolayer. The bacterial strain cytotoxicity was assessed by counting the eukaryotic viable cells after the infection with the different strains and calculating the percentage of mortality as stated before. The different bacterial strains were grouped according their capability to form IBC, IIB, or none. No relationship was observed among levels of citotoxicity and intracellular lifestyle. Statistical: Mann-Whitney non-parametric test, p ≤ 0.05 was considered significant. The assay was performed in triplicate.

*E. coli* strains exerted variable cytotoxicity on the cultured cells (0 to 90%), although most of the strains showed intermediate values. Three strains (208, 191, and 234) showed a cytotoxic effect higher than 75%, and the other 3 (7, 156, and 221) did not induce cell death (**Figure 3**). Two of the three strains that showed the highest cytotoxicity (more than 75%) had the cnf1 gene (which could express the CNF1 toxin and act on the urothelial cells).

### Intracellular Bacteria Localization by Laser Confocal Microscopy

Although all the evaluated UPECs were able to enter the eukaryotic cells, confirmation and localization of intracellular bacteria was performed by confocal laser scanning microscopy (CLSM). Four strains were tested, two of which were negative for the presence of IBC in urine desquamated cells (Robino et al., 2014).

**Figure 4:**
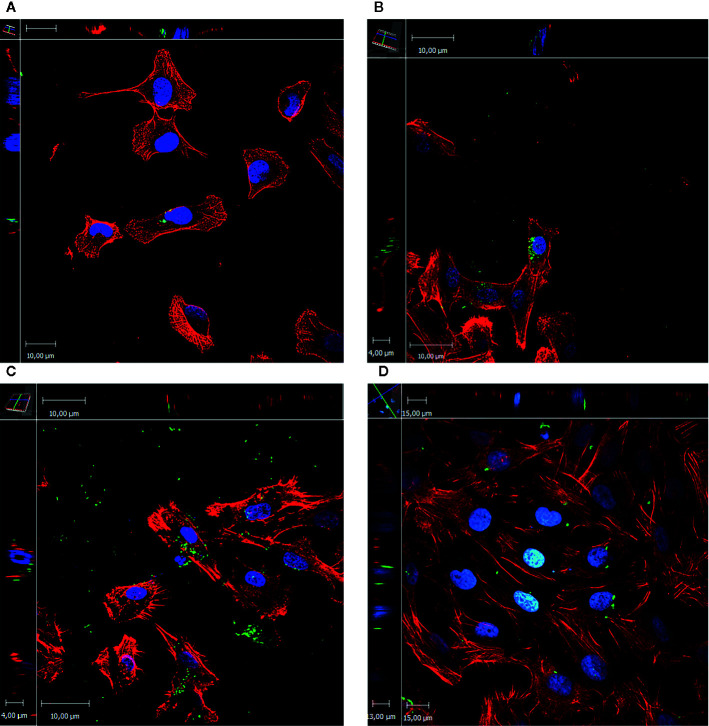
*E*. *coli* invasion assay in T24 cells. The images represent the xyz stacks obtained with CLSM. Maximum intensity z-projections are shown in the central panel, upper and left are zx and zy, respectively. In red actin staining (rhodamine-phalloidin), in blue DNA (Hoescht 333452), and in green UPEC (antibody against *E*. *coli* coupled to FitC). **(A)** UPEC 7, the intracellular bacteria are observed in groups inside the eukaryotic cell resembling IBC. **(B)** UPEC 144, intracellular bacteria are observed in big groups in a perinuclear localization. **(C)** UPEC 172, in this case the intracellular bacteria are dispersed in the cytoplasm of the eukaryotic cell. Severe damage is observed as the cells had their membrane damaged and the presence of philopodia this is in agreement with cytotoxicity assay as UPEC 172 had one of the highest values. **(D)** UPEC 174, disperse intracellular bacteria is observed.

After image acquisition and their subsequent analysis, all the strains were observed in small groups, rather than dispersed in the cytoplasm (**Figure 4**).

### Antibiotics Effect on Intracellular Bacteria

In order to evaluate the effect of antibiotics on intracellular bacteria, the following antimicrobials were used: ceftriaxone (500, 1,000, and 1,500 µg/ml); amikacin (500, 1,000, and 2,000 µg/ml); ciprofloxacin (2, 3, and 5 µg/ml), and azithromycin (20, 100 and 500 µg/ml). Strains 7 and 144 were selected for these assay because they showed different invasion percentages (confirmed by confocal microscopy) and low cytotoxicity.

**Figure 5:**
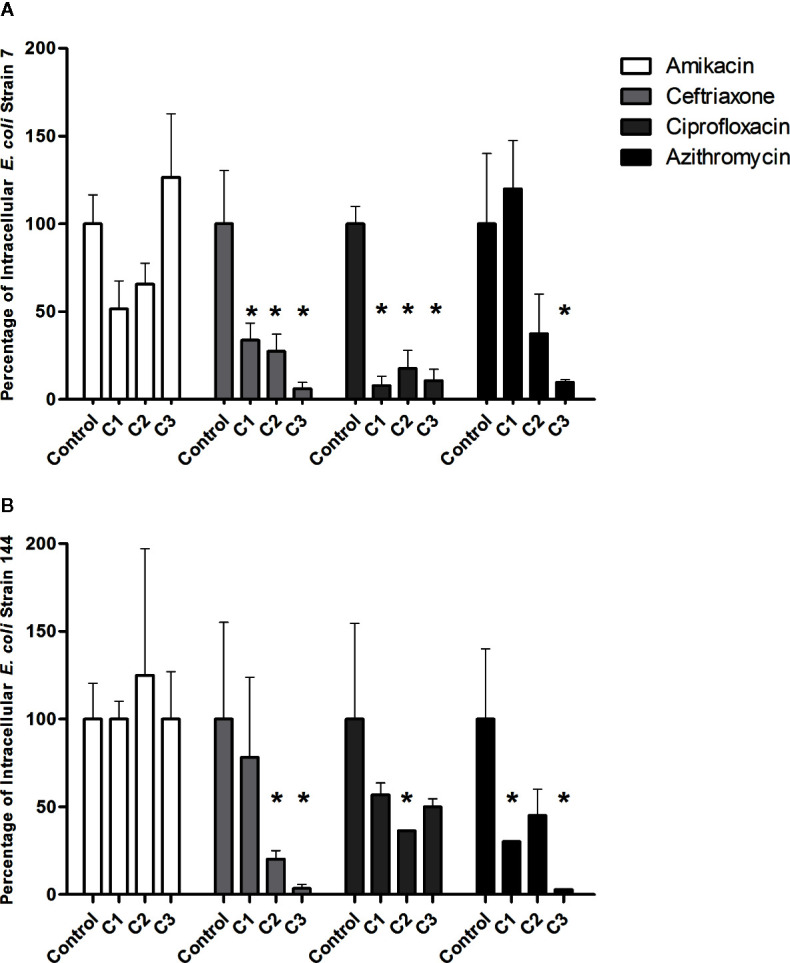
Percentage of intracellular bacteria after antibiotic treatment. **(A)**, strain 7. **(B)**, strain 144. The values obtained after infection without antibiotics was the control and was set as 100%. After each treatment, the percentage of surviving intracellular bacteria was calculated according to the control. The asterisk represents the cases were significant values were obtained compared with each control (P < 0.05). Statistical: Mann-Whitney non-parametric test. The assay was performed in triplicate.

Amikacin, which belongs to the group of aminoglycosides and is not able to enter the eukaryotic cells, did not generate a significant effect on the percentages of intracellular bacteria (**Figure 5** panel A and B). Regarding ceftriaxone, a significant decrease in intracellular bacteria percentage was observed at the three concentrations tested 500,1,000, and 1,500 µg/ml for strain 7 and at 1,000 and 1,500 µg/ml for the strain 144 (**Figure 5**).

Ciprofloxacin is the only antibiotic used for the UTI treatment, which achieves high intracellular concentrations. A significant decrease of intracellular bacteria was observed for strain 7 after all ciprofloxacin concentrations. On the other hand, for strain 144, the significant decrease was observed only with 3 µg/ml of ciprofloxacin (**Figure 5**).

Concerning the effect of azithromycin, a significant decrease in the percentage of intracellular bacteria was observed for both isolates at the highest concentration (500 µg/ml) and 20 µg/ml for 144 (**Figure 5**).

Amikacin C1: 500 µg/ml, C2: 1,000 µg/ml, C3: 2,000 µg/ml; Ceftriaxone: C1: 500 µg/ml, C2: 1,000 µg/ml, C3: 1,500 µg/ml; Ciprofloxacine C1: 2 µg/ml, C2: 3 µg/ml, C3: 5 µg/ml; Azithromycin: C1 20 µg/ml, C2: 100 µg/ml, C3: 500 µg/ml.

### Cytotoxicity of Antibiotics on Cultured Cells

**Figure 6:**
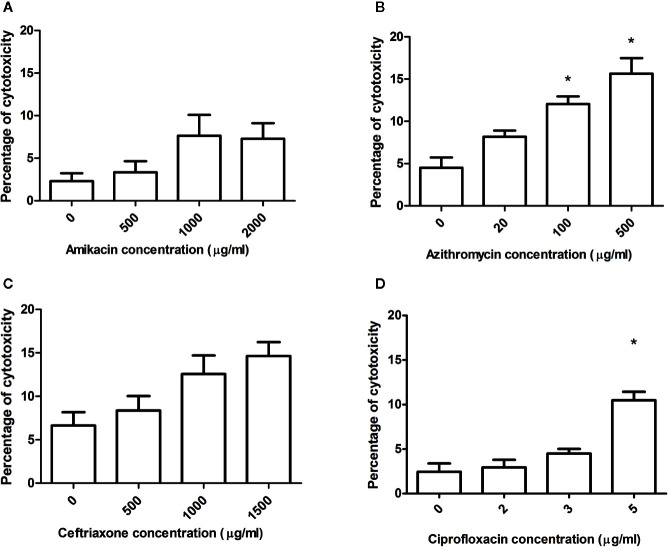
Antibiotic cytotoxicity over the cell monolayer. Each antibiotic concentration was evaluated on the monolayer during 10 h of incubation. Using MLC, the total number of cells per field and the number of dead cells was observed, and the percentage was calculated. **(A)** Cytotoxicity for the different concentrations of amikacin (AK), **(B)** azithromycin (AZ), **(C)** ceftriaxone (CRO) and **(D)** ciprofloxacin (CIP). The results were compared using the Mann-Whitney statistical test, and it was considered significant when the p-value ≤ 0.05. *Significant differences respect to control.

To rule out if the decrease of infection was due to antibiotics cytotoxicity, the cells were exposed to antibiotics for 10 h. Then, cytotoxicity was assessed using a cell’s differential staining (live and dead) and CLSM analysis of images. It was observed in general that cytotoxicity increased as the concentration of the antibiotic augmented, but it never exceeded 20% (**Figure 6**). Amikacin and ceftriaxone did not produce significant cytotoxicity of the cells when compared with cells without antibiotics in any concentration. In the case of azithromycin, a significant increase in the cytotoxicity was observed for the highest concentrations (100, 500). Lastly, a significant increase in cytotoxicity was observed when the highest concentration of ciprofloxacin was used.

In order to study if the increase in cytotoxicity produced by antibiotics, is contributing to the reduction of the intracellular bacteria we have performed a correlation study (Pearson coefficient and correlation matrix) only in the cases that significant increase in cytotoxicity was observed. When we correlated the antibiotic concentration, the percentage of intracellular surviving bacteria and cytotoxicity we observed that for ciprofloxacin: the increase of the antibiotic concentration significantly correlated with the decrease of the intracellular surviving bacteria (P = 0,0146, r = -0,55), the increase of the antibiotic concentration significantly correlated with the increase in cytotoxicity (P = 0,001, r = 0,82), but the correlation matrix between this two-parameter was not statistically significant (P = 0,126). The only case where we observed that the reduction of the surviving intracellular bacteria was explained by the increase in cytotoxicity was in the case of strain 7 with the highest concentration of azithromycin (P = 0,025). With the values obtained with the strain 144, we did not observe the same correlation (P = 0,131). As a result of the Pearson correlation, we can affirm that the only case that the antibiotic cytotoxicity account in the reduction of the intracellular survival bacteria was in the case azithromycin (500 ug/ml) with the strain 7. In all the other cases we were not able to observe a significant correlation between the percentage of intracellular bacteria and the percentage of cytotoxicity.

## Discussion

UPEC is the principal etiological agent associated with community-acquired UTI (Ronald, 2003). One of the mechanisms used by UPEC to evade the immune response and the action of the antibiotic is to invade and form IBC in the superficial bladder cells (Anderson et al., 2003). IBC in humans was first described in desquamated bladder cells of the urine of women with cystitis (Rosen et al., 2007). Then, our group described IBC in 36,8% of children with UTI (Robino et al., 2013; Robino et al., 2014), and their presence was related to UTIs recurrency (Robino et al., 2014).

So far, it is not known if all clinical uropathogenic *E. coli* can enter the urothelial cell and develop IBC. Most of the studies that report *E. coli* ability to form IBC have been performed using desquamated bladder epithelial cells in urine or in vitro or mouse UTI model using a few highly characterized isolates, such as UTI89 (Anderson et al., 2004; Garofalo et al., 2007; Rosen et al., 2007; Robino et al., 2014). For this reason, 23 strains that had been previously classified according to the presence/absence of IBC/IIB in desquamated bladder cells in urine from children with UTI and that have a great diversity of virulence factor profiles (Robino et al., 2014) were selected for this study. In this work, it was observed that all the strains were able to invade epithelial cells in vitro, showing a very variable intracellular bacterial survival percentage. The percentage of viable intracellular bacteria and the previous presence of IBC or IIB in the patient’s desquamated urine cells were not related. This finding suggests that the absence of IBC in urothelial cells in the urine may be due to other factors independent of the bacteria. IBC formation stages are transiet during an UTI episode, and it does not occur in all cells at the same time, so the presence or absence of IBC in bladder desquamated urine cells could depend on the moment the sample is collected and the volume. Other factors could be related to the host and the immune response (Bien et al., 2012).

Garofalo et al. studied 18 UPEC clinical isolates from women with UTI in a murine model looking for IBC formation (Garofalo et al., 2007). They found that most of the strains were able to form IBC, and those that were not capable did so when co-infecting with an IBC-forming strain. These results support the hypothesis that although UPEC is capable of generating infection in a cell culture model, there are specific factors related to the host that affect the ability to form IBC in vivo.

The isolates studied had been previously classified as biofilm- or non-biofilm-producers (González et al., 2017). The ability to form biofilm was positively associated with higher percentages of intracellular bacteria in this work. Several authors have proposed that IBC are similar to biofilms since they have an extracellular polysaccharide matrix, they go through several stages of maturation, and phenotypic changes in the bacteria are seen (Mulvey, 2002). Many of the virulence factors that allow UPEC to form biofilm also participate in the adhesion and invasion of urothelial cells (Flores-Mireles et al., 2015), such is the case of FimH.

Four strains were chosen to confirm the localization within the eukaryotic cells: 2 of them previously classified as negative IBC/IIB in desquamated bladder cells from urine, 1 IBC producer, and the other one classified as IIB (Robino et al., 2014). The four strains invade the cells and localize in groups near the nucleus.

The mechanism of UPEC to form IBC or persist in the bladder epithelium as QIRs could explain the recurrence of infection after the treatments of UTI with antibiotics (Schilling et al., 2002; Justice et al., 2004). Most of the antibiotics are not able to enter the cells, except quinolones, such as ciprofloxacin. For this reason, to evaluate if intracellular bacteria localization acts as a barrier for antibiotic activity, we tested some antibiotics that are frequently used for UTI treatment, at the concentrations achieved in the urine. Antibiotic cytotoxicity over the cell monolayer was also evaluated. Only two antibiotics (ciprofloxacin 5 µg/ml and azithromycin 100 and 500 µg/ml) induced significant cytotoxicity in T24 cells compared with cells without antibiotics. Ciprofloxacin commonly enters host cells, and it was the only antibiotic that exerted a significant bacterial decrease at the three concentrations tested in vitro. Even though an increase of the antibiotic concentration significantly correlated with a decrease of the surviving intracellular bacteria and an increase in cytotoxicity, the correlation matrix between these two parameters was not statistically significant, concluding that antibiotic cytotoxicity by itself does not affect intracellular bacterial survival.

Ceftriaxone, a third-generation cephalosporin, is not classified as an antibiotic with intracellular activity. However, at high concentrations, a significant decrease in the percentage of intracellular bacteria was observed, without cytotoxicity over the culture cells.

Blango et al. (2010) studied the effect of different antibiotics on in vitro IBC and ex vivo using a murine model (Blango and Mulvey, 2010). They observed that nitrofurantoin and quinolones like ciprofloxacin decreased the percentage of intracellular bacteria in vitro. However, these antibiotics were not able to eradicate intracellular bacteria in the bladder tissue of mice (Blango and Mulvey, 2010).

Azithromycin is a macrolide with high intracellular activity, usually indicated in respiratory or genital infections caused by Mycoplasma or intracellular bacterias like Chlamydia (Parnham et al., 2014). It is not recommended for UTI treatment because it reaches a low urine concentration. In this study, azithromycin induced a significant decrease in intracellular bacteria percentage at 500 µg/ml, and also exerted high cytotoxicity. Even though azithromycin does not reach a high urine concentration after an oral dose, this data would be relevant in order to evaluate if there are any other administration routes to the patient (maybe locally or the development of antibiotic nano delivery systems) to achieve adequate concentrations. Studying the effect of combined antibiotic treatment, like a beta-lactam (frequently used for UTI treatment) and azithromycin, could be an alternative for recurrent UTI treatment where intracellular bacteria could be involved.

Future work should evaluate the use of these and other antibiotics using primary cultures and *in vivo* models in order to consolidate these data.

## Conclusions

All the UPEC clinical isolates could invade bladder epithelial cells in vitro, and those analyzed by confocal microscopy showed to be in small clusters near the nucleus. Ceftriaxone, ciprofloxacin, and azithromycin could reduce the percentage of intracellular bacteria in vitro. In vivo and case-control, studies should be performed in order to evaluate which antibiotic therapy is the best for intracellular bacteria and to reduce recurrent infections.

## Data Availability Statement

All datasets generated for this study are included in the article/supplementary material.

## Author Contributions

MG, PS, and LR contributed to the design and experimental work. All authors contributed to the article and approved the submitted version.

## Conflict of Interest

The authors declare that the research was conducted in the absence of any commercial or financial relationships that could be construed as a potential conflict of interest.
